# Fabrication of SrTiO_3_ anchored rGO/g-C_3_N_4_ photocatalyst for the removal of mixed dye from wastewater: dual photocatalytic mechanism

**DOI:** 10.1038/s41598-024-66844-x

**Published:** 2024-07-15

**Authors:** Venkatesh Gopal, Govindasamy Palanisamy, Jintae Lee, Imad A. Abu-Yousef, Amin F. Majdalawieh, Amjad Mahasneh, Kattupatti M. Prabu, Sofian Kanan

**Affiliations:** 1 Department of Biology, Chemistry and Environmental Sciences, College of Arts and Sciences, American University of Sharjah, P.O. Box 26666, Sharjah, United Arab Emirates; 2 School of Chemical Engineering, Yeungnam University, 280 Daehak-Ro, Gyeongsan, 38541 Republic of Korea; 3 PG and Research Department of Physics, Sri Vidya Mandir Arts and Science College, Katteri, Uthangarai, Tamilnadu 636 902 India

**Keywords:** Photocatalytic, Reduced GO, SrTiO_3_, Methylene blue, Rhodamine B, Wet impregnation, Photocatalysis, Materials for energy and catalysis, Techniques and instrumentation, Environmental sciences

## Abstract

A metal-free combination of rGO/g-C_3_N_4_-coupled SrTiO_3_ (SRN) ternary nanocomposite prepared via a wet impregnation method for UV–Vis light photocatalytic applications. Various physicochemical properties of the samples were investigated by several spectroscopic techniques including X-ray diffraction (XRD), FT-IR, Raman, field emission scanning electron microscopy with energy dispersive X-ray spectroscopy (FE-SEM-EDX), high-resolution transmission electron microscopy (HR-TEM), UV–Vis, photoluminescence (PL), X-ray photoelectron spectroscopy (XPS) and Brunauer–Emmett–Teller (BET) surface area analysis. The data suggest agglomerated SRT nanoparticles are dispersed and distributed throughout the surface of the rGO sheets and GCN nanostructures. The photocatalytic performance of the SRN towards combined mixed dye and its degradation activities were evaluated towards the most common industrial effluents, Rhodamine B (RhB) and Methylene blue (MB), under UV–Vis light illumination. The results revealed that the degradation efficiency of the SRN photocatalyst shows excellent performance compared with that of the binary composition and the pure SrTiO_3_ (SRT) sample. The reaction rate constant for RhB was estimated to be 0.0039 min^−1^ and for MB to be 0.0316 min^−1^, which are 3.26 (RhB) and 4.21 (MB) times faster than the pure SRT sample. The enhanced degradation efficiency was attained not only by interfacial formation but also by the speedy transportation of electrons across the heterojunction. After 5 runs of the photocatalytic recylic process, the SRN photocatalyst exhibited ultimate stability without structural changes, and no noticeable degradation was observed. The outcomes of the ternary SRN nanocomposite manifest a dual photocatalytic scheme, the photocatalytic enrichment could be caused by the Z-scheme charge transfer process between GCN, SRT, and rGO nanocomposite, which helps effectual charge separation and keeps a high redox potential. From the results, SRN sample provides insight into the integration of an effective and potential photocatalyst for wastewater treatment toward real-time environmental remediation applications.

## Introduction

The abrupt increase in modern technology, industrialization, human population, and atmospheric climate variation severely affects the environment. In specific, the manufacturing industries of textile dyes, cosmetics, leather, and paper discharge of the contaminants directly to the ecology system causes serious environmental problems^1^. Organic dyes are toxic pollutants that provoke negative effects on human health^2^. To overcome such a problem researchers have followed several strategies to eliminate such pollutants from waste water^3,4^. Among them, the photocatalytic dye degradation process garnered attention because of its obvious advantages like the ease to operate, simple instrumentation, non-selective oxidation, cost efficiency, renewability, and eco-friendliness^5^. So far various photocatalysts have been suggested in specific fields of applications to solve this problem^6,7^. However, they show some limitations like wide bandgap, very expensive, inappropriate band positions, and poor degradation efficiency^8–11^. To resolve the above discussed bottlenecks an effective, cost efficient, environment friendly, and non-toxic semiconductor photocatalysts are necessary.

Recently, ABO_3_ type titanate perovskites ATiO_3_ (A = Ca, Sr, Ba, etc.) received great attention with its advantageous properties like superior electronic, optical, magnetic, and excellent photocatalytic properties in the UV–Vis region^12–14^. SrTiO_3_ has proven to be an outstanding option to design and explore a new kind of promising material for photocatalytic application^15,16^. Moreover, researchers interest in SrTiO_3_ has been sparked by its extraordinary physicochemical properties, such as excellent thermal and structural stability, photo corrosion reliability, as well as, an increased number of photocatalytic active sites, which makes these material desirable for various applications^17,18^. Some pitfalls of SrTiO_3_. such as larger bandgap, poor absorption in visible light, and low stability, however, make it not suitable for practical photocatalytic applications.

In order to rectify the aforementioned obstacles, alternative and extensive research is essential to enhance the photocatalytic performance by tunning the optical band gap and other properties. Therefore, several modifications of SrTiO_3_ by doping/coupling with other semiconductor metals, non-metals, and carbon-based materials have been reported. Recently, carbon and graphene-based nanocomposites have been reported to have exceptional semiconductor properties in multifield applications^19–21^. Graphene possess 2D structure with remarkable features like high electrical and thermal conductivity, surpassing electron transportation ability, mechanical strength, and chemical stability^22,23^. In addition, several research groups have explored the use of graphitic carbon nitride (g-C_3_N_4_) to establish an enhanced transfer of photoinduced electrons to improve the photocatalytic activity of materials. It is notable visible light photocatalyst due to its optimal energy bandgap (~ 2.71 eV). The conduction band (CB) bottom of g-C_3_N_4_ is positioned at approximately − 1.1 eV (vs. normal hydrogen electrode [NHE], pH 0). This high CB position grants the CB electrons of g-C_3_N_4_ a strong reduction capability, making it an exemplary reduction photocatalyst^24^. Moreover, g-C_3_N_4_ received attention because of its medium band gap, cost effectiveness, facility to synthesis, high chemical stability and non-toxicity^25,26^. g-C_3_N_4_ photocatalysts have attracted significant attention for a variety of photocatalytic applications, such as hydrogen evolution, CO_2_ reduction, H_2_O_2_ production, and pollutant degradation, among others^27,28^. Therefore, the combination of graphene with semiconductors can effectively promote the electron transporting between the junctions, and thus enhance the catalytic chemical reactions^29^. The use of rGO/g-C_3_N_4_ coupled SrTiO_3_ nanomaterials for the removal of organic dyes was very rarely reported. For example, Rosy et al.^30^ investigated the performance of rGO/SrTiO_3_ nanocomposite over Rhodamine B, and Rose Bengal (RB) by dye degradation process. He et al.^31^ evaluated the SrTiO_3_/graphene composite for the degradation of RhB pollutant under UV light irradiation. Luo et al.^32^ reported that the combination of g-C_3_N_4_ and SrTiO_3_ could enhance the photocatalytic performance. Furthermore, Cr-doped, N-doped SrTiO_3_/g-C_3_N_4_ and g-C_3_N_4_/SrTiO_3_ heterostructures were, also, studied for photocatalytic based environmental remediation applications^33–35^. Furthermore, several strategies like doping and coupling with metal, and non-metal compositions have been utilized to improve the properties in photocatalytic applications^36,37^. Hence, the development of highly efficient materials by facile, time-consuming, green, and efficient synthetic route for photocatalytic applications are extremely anticipated. Furthermore, the charge transfer process by S-scheme and Z-scheme charge carrier pathways are prominent for degradation activity. S-scheme photocatalytic process involves a step-scheme mechanism that enhances charge separation by utilizing a staggered band alignment, improving the overall photocatalytic efficiency^38–40^. However, S-scheme has a drawback of low efficiency due to the potential recombination of photo-generated electron–hole pairs, which reduces the overall photocatalytic performance. In contrast, the Z-scheme photocatalytic process offers a significant advantage by mimicking natural photosynthesis, effectively separating the electron–hole pairs, and thereby enhancing the photocatalytic efficiency. This separation results in improved charge carrier dynamics and a greater potential for driving redox reactions, making the Z-scheme a more efficient and effective photocatalytic system^41,42^.

In this work, the synergistic effect of the prepared heterostructure SRN nanocomposite is thoroughly analyzed by various characterization techniques. The assessment of photocatalytic performance was investigated by the degradation of a mixed dye (MB + RhB), under UV–Vis light irradiation. The stability, recyclability, radicals’ responsibility in the degradation process have been studied. Furthermore, the possible photocatalytic Z-scheme charge transfer mechanism is also discussed and proposed by schematic representation.

## Experimental section

### Materials

All the analytical grade (AR) reagents were purchased from Merck in India and Loba chemicals. All the chemicals were directly used without purification. Titanium (IV) isopropoxide (C_12_H_28_O_4_Ti)—97% (TTIP), Strontium chloride (SrCl_2_)—99.99%, Potassium Hydroxide (KOH)—97%, Ethyl alcohol (C_2_H_6_O)—99.9%, Melamine (C_3_H_6_N_6_), Graphite powder—99.5%, Hydrogen peroxide (H_2_O_2_)—30% purity, Sulfuric acid (H_2_SO_4_)—97%, Hydrochloric acid (HCl)—38.0%, Potassium permanganate (KMnO_4_)—99.0%, Sodium nitrate (NaNO_3_)—99.0%, and De-ionized (DI) water were used for all the synthesis process. Methylene Blue (C_16_H_18_ClN_3_S) (MB) and Rhodamine B (C_28_H_31_ClN_2_O_3_) were selected as the target organic pollutants.

### Preparation of reduced graphene oxide (rGO), SrTiO_3_ (SRT) and rGO/SrTiO_3_ (SRG) nanocomposites

The SrTiO_3_ (SRT) was prepared using co-solvent synthetic process via simple hydrothermal approach. Initially, ethanolic solution of 1.77 g of TTIP was taken and stirred for 1 h at room temperature. Secondly, 1.47 g of SrCl_2_ and required quantity of KOH were dissolved in 10.0 mL of DI water in separate beaker. Then the prepared solutions were mixed dropwise to the initial solution under stirring process for 30 min. After the suspension was transferred to a stainless-steel hydrothermal vessel and kept in an oven for 4 h at 200 °C. Then the vessel was allowed to auto cool, the resultant suspension was carefully washed with acetic acid and DI water several times using centrifugation process. Finally, the obtained products were dried in an hot air oven at 80 °C for overnight^43^.

Reduced graphene oxide, SrTiO_3_, and rGO/SrTiO_3_ were prepared via wet impregnation method as was previously reported^43^. In brief, an appropriate amount of GO was mixed with an equal ratio of ethanol and DI water, then it was dispersed for 2 h using ultrasonication. The prepared 1.0 g SrTiO_3_ was then added in a GO solution and stirred for 2 h at room temperature to obtain a homogeneous suspension. Then the mixture was transferred into a hydrothermal autoclave and placed in a hot air oven at 120 °C, for 12 h. The vessel was allowed to auto cool and then the suspension was centrifuged and washed with DI water and ethanol several times. Finally, the obtained product was dried in a hot air oven for 12 h at 80 °C.

### Synthesis procedure for g-C_3_N_4_ (GCN) and SrTiO_3_/rGO/g-C_3_N_4_ (SRN)

To obtain GCN nanosheets, 5.0 g of melamine powder in a silica crucible, with cover, was placed in a muffle furnace at 540 °C for 2 h, and subsequently allowed to naturally cooldown. The obtained bulk, in yellow precipitate form, was then crushed and treated with hydrochloric acid (HCl—15 wt.% in 50 mL DI water)^44,45^. Then the obtained sample was further centrifuged and washed with DI water five times. Then it was dried overnight in a hot air oven at 80 °C.

The synthesis of SRN was done by the wet impregnation method where a 2:1 ratio of SRG and GCN was dispersed and mixed with water/ethanol solution by ultrasonication. Then the products were filtered and dried overnight in a hot air oven at 80 °C.

### Material characterization

The prepared samples were analyzed using a Rigaku D/Max Ultima III X-ray diffraction instrument to determine the (XRD) patterns. Functional groups of the samples were identified by a Fourier transform infrared (FT-IR) NEXUS 470 spectrometer. Ultraviolet–visible (UV–Vis) absorption spectra were obtained by a JASCO V-770 spectrophotometer. The surface morphology and microstructure of the prepared products were analysed using FESEM Hitachi S-4800, HRTEM, and EDX JEOL JEM 2100 instruments. The different chemical states and electronic structure of the samples were identified by X-ray photoelectron spectroscopy XPS, ESCA 3400 spectrometer. The charge recombination process in the samples was detected by photoluminescence (PL) spectra JASCO-spectrofluorometer FP-8200. Raman analysis from Confocal Raman Microscope (WiTec alpha 300, Germany), particle size analysis was performed on dynamic light scattering (DLS) analysis Particle analyser lite sizer 500 (Anton Paar). Nitrogen adsorption and desorption isotherms were measured at 77 K using “Autosorb iQ” from Quantachrome. Prior to the experiment, the samples were outgassed at 393 K and under a vacuum of 10^−6^ torr to constant pressure. Characterization of pore sizes and pore structure were accomplished using density functional theory (DFT). Moreover, Brunauer–Emmett–Teller (BET) theory was used to calculate the BET surface areas (*S*_BET_).

### Evaluation of adsorption and photocatalytic performance

The photocatalytic ability of the prepared samples was examined by a mixed dye (MB + RhB) degradation under UV–Vis light irradiation (500 W Halogen lamp). In brief, a 100 mg of photocatalysts was mixed in 100 mL of MB and RhB 10 ppm of aqueous solution was taken in a double-layered beaker and stirred for few minutes. The prepared mixtures were kept in a dark area for 1 h to attain an adsorption/desorption equilibrium condition. Initially, 1 mL of aliquots was pipetted out, prior to the irradiation and then the aqueous solution was irradiated. The irradiation source was kept at a 10 cm distance from the solution. In a typical photocatalytic experiment, after irradiation starts, 1.0 mL of aliquots was pipetted out for every 20 min of time intervals. Then, the aliquots were centrifuged to remove the catalysts in the solution. The dye concentration after the photocatalytic experiments was examined using UV–Vis absorption spectrophotometer. The same procedure was followed for evaluating radicals, stability, and the recycle test under similar conditions. To determine the scavengers responsible for degradation, 1 mM of Benzoquinone (BQ—98%) for superoxide radicals (O_2_^·−^), Ammonium oxalate (AO—99%) for holes (h^+^) and Isopropyl alcohol (IPA—99.8%) for hydroxyl radicals (OH^·^) were used as precursors in similar photocatalytic radical trapping experiments.

## Results and discussion

### XRD analysis

**Figure 1:**
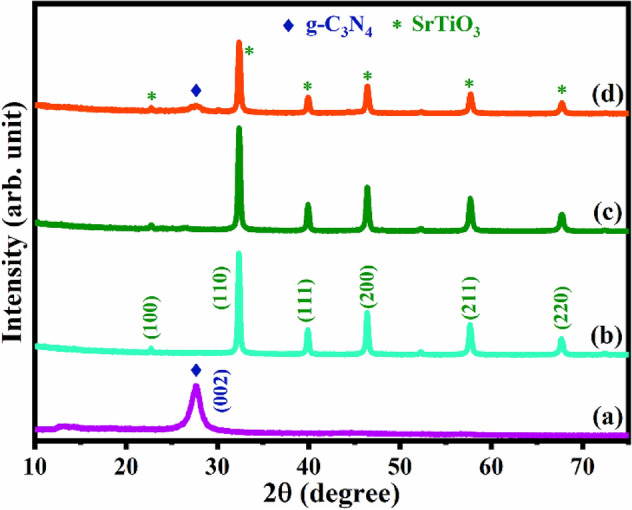
XRD patterns for (**a**) GCN, (**b**) SRT, (**c**) SRG, and (**d**) SRN nanocomposite.

The crystallographic structure and phase purity of the prepared samples were examined by XRD analysis. Figure 1 presents the recorded diffraction peaks of pure and composite samples. In the case of pure GCN the characteristic peak, obtained at 2θ value = 27.5° (002) plane, is well matched with JCPDS file 87-1526 ^46^ of g-C_3_N_4_, is shown in Fig. 1a. As shown in Fig. 1b, the observed peaks indicating the perovskite-type cubic phase structure of SRT at angles 22.5, 32.1, 39.6, 46.1, 57.3 and 67.2° correspond to (100), (110), (111), (200), (211) and (220) planes, respectively (JCPDS No. 40-1500)^43^. Figure 1c shows the XRD peaks of the SRG nanocomposite sample with similar diffraction peaks of SRT. However, no peaks were observed for rGO in the SRG nanocomposite due to weak diffraction intensity and the low amount of rGO introduced in the resultant product^15,47^. Figure 1d shows the distinct XRD diffraction peaks of GCN and SRT (marked with different symbols), evidently appearing in the SRN sample with no other impurities peaks, which confirms the successful formation of a ternary nanocomposite. Moreover, the implementation of GCN and rGO in the SRT sample varies/decreases the XRD peak intensities which may be due to the influence of GCN and rGO sheets being successfully deposited on the SRT sample. Thus, the synthesised SRN nanocomposite strongly shows the interaction between heterostructure formation of ternary nanocomposite during the preparation process.

### FT-IR analysis

**Figure 2:**
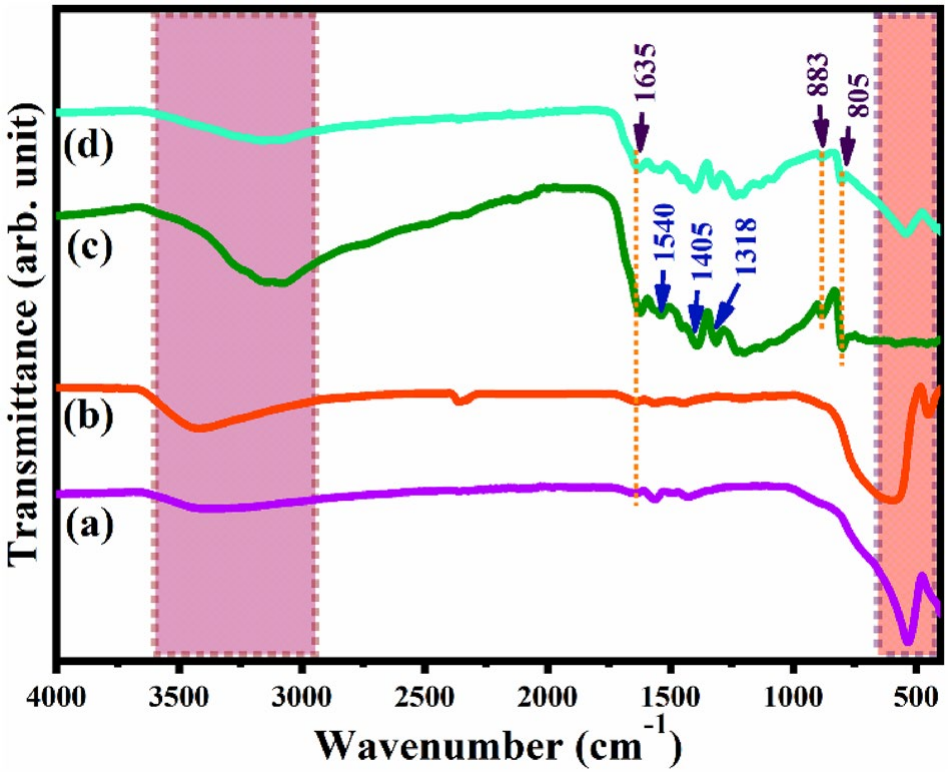
FT-IR spectra for (**a**) SRT, (**b**) SRG, (**c**) GCN, and (**d**) SRN nanocomposites.

The investigation of functional groups present in the prepared samples was conducted by FT-IR analysis. Figure 2 shows the FTIR spectra for bare GCN, SRT, SRG and SRN nanocomposite. As shown in Fig. 2a, the strong peaks, appearing between 550 and 450 cm^−1^, may be attributed to stretching and bending mode vibrations of Ti–O bond and O–Ti–O bonds respectively, in the presence of TiO_6_ octahedron^48^. Furthermore, the sharp peak exhibited at 545 cm^−1^ in all the FT-IR spectra corresponds to M–O (metal–oxygen bond)^49^. For SRG sample, as shown in Fig. 2b, the presence of rGO in the sample, ascribed by the peak at 1446 cm^−1^ , is due to C–H bending vibration, and the peak obtained at 1210 cm^−1^ corresponds to C=C asymmetric stretching vibration^50^. From Fig. 2c, the small peaks appearing in the range between 1210 to 1650 cm^−1^ are attributed to stretching mode vibrations of C–N and C=N heterocyclic group of graphitic carbon nitride. Furthermore, A small sharp peak observed at 883 and 805 cm^−1^ was attributed to the breathing mode of tri-s-triazine ring^51–53^. For all the spectra in Fig. 2, the broad peak observed in the region 3500–3000 cm^−1^ indicates the O–H stretching vibration and surface absorbed water molecules. After the introduction of GCN in the SRG sample the SRN peaks were weaker than those for pure GCN and the SRT sample reveals the successful heterostructure formation of ternary nanocomposite. Moreover, all the characteristic peaks of GCN and rGO are obviously exhibited in the FT-IR spectra of SRN nanocomposite.

### Raman analysis

The Raman analysis for SRT, rGO, GCN, and SRN samples were carried out with an excitation wavelength 780 nm with all recorded spectra are presented in Fig. S1. The characteristic peaks of GCN appeared at 478, 710, 760, 984, 1239 and 1313 cm^−1^ are corresponding to the Raman spectrum of the pure g-C_3_N_4_. The spectra ascribed between the range from 1000 to 1700 cm^−1^ is owing to g-C_3_N_4_ layer formation and the symmetric sp^2^ stretching mode of graphitic nature^54,55^. In the case of rGO in Fig. S1, the two different peaks appeared at 1352 and 1583 cm^−1^ confirms the D and G bands of rGO, which denotes the E_2g_ stretching mode of sp^2^ bond and G band denotes A_1g_ carbon vibration modes, respectively^56^. The vibration modes of SRT ascribed with different characteristic positions at 183 cm^−1^, 245–352 cm^−1^, 542 cm^−1^, 625–721 cm^−1^ and 798 cm^−1^ are affirmed the presence of TO_2_ (O–Sr–O) bending, TO_3_ (O–Sr–O) stretching mode, TO_4_ (O–Sr–O)/, TO (Ti–O–Ti) bending mode, and TO_4_ (Ti–O) stretching modes, respectively^57–59^. From Fig. S1, the similar peaks of SRT, rGO and GCN confirm the presence in ternary system, indicative of the successful formation of the heterostructured SRN nanocomposite. Even though, the characteristic peaks of rGO and GCN are difficult to identify in the Raman spectrum of SRN composite, indicating the high dispersion/aggregation of g-C_3_N_4_ and rGO nanoparticles on the surface of the composite ^60^.

### Morphological analysis

**Figure 3:**
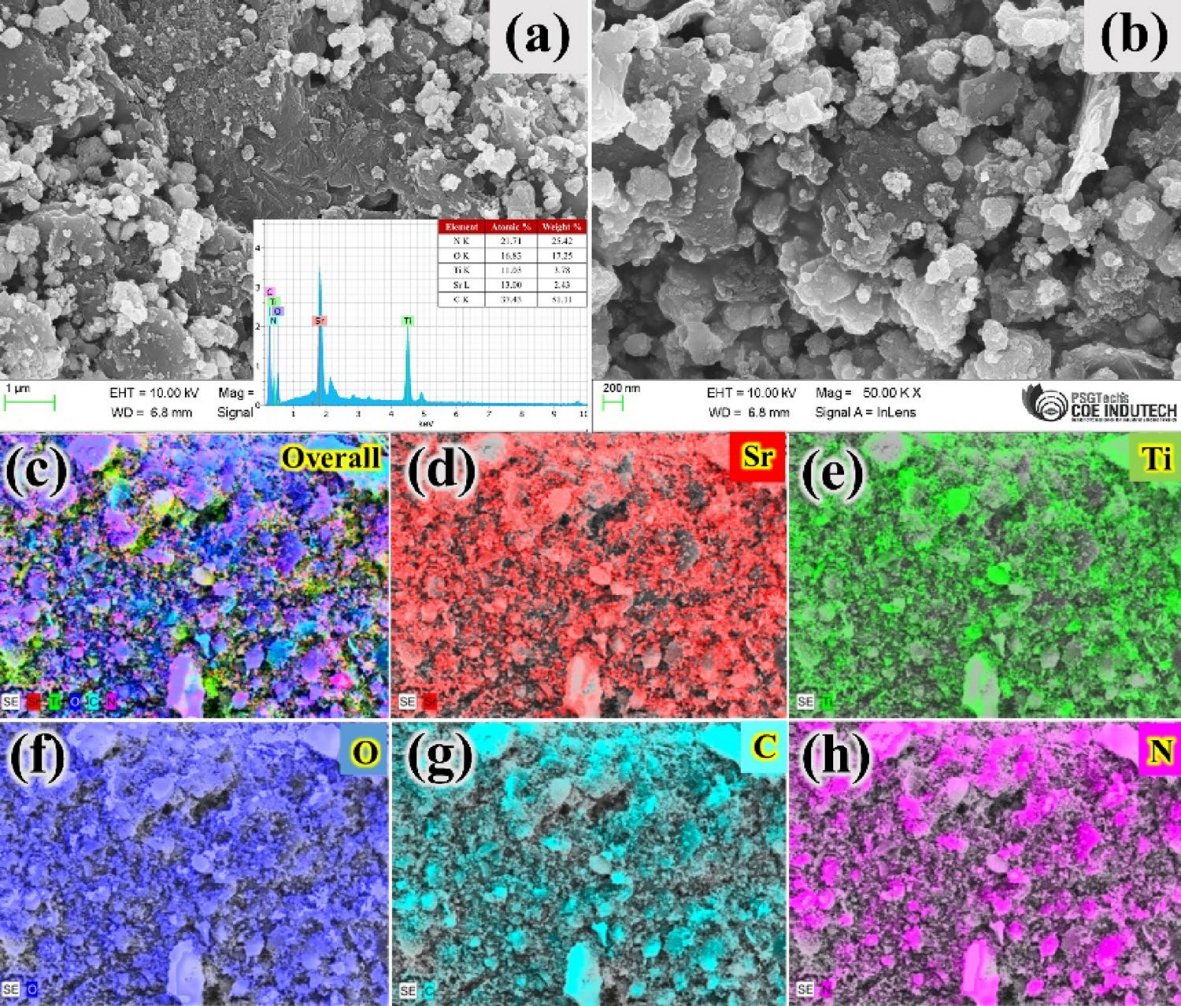
(**a**) and (**b**) FESEM images, (inset) EDS spectrum and (**c**–**h**) elemental mapping distribution of SRN nanocomposite.

The micrographs and surface morphologies of the SRN sample were investigated by FESEM analysis, and the results can be visualized in Fig. 3a,b. The inset in Fig. 3a shows the EDS spectra of different elements present in the SRN sample. As shown in Fig. 3a,b, it can be observed that the SRT samples are seriously agglomerated and randomly distributed over the surface of GCN and rGO nanostructures. Notably, the SRT particles are closely aggregated and intercalated with the GCN surfaces, and the rGO sheets might well be responsible for promoting the effective photocatalytic activity^61^. As shown in Fig. 3a,b, the rough surfaces observed on the particles are due to the utilization of low viscosity aqueous solvent medium, which is responsible for the agglomeration of nanoparticles. In low viscosity solvents, there is a high rate of diffusion, enabling nanostructures to grow at a faster rate^62,63^.

The various elements obtained in the SRN nanocomposite were revealed by EDS analysis and the corresponding results are displayed in the inset of Fig. 3a. The elements of Sr, Ti, O, N and C that exist in the SRN nanocomposite indicates a successful formation, and that no other impurities were detected in the prepared sample. Furthermore, the uniform distribution of elements throughout the prepared nanocomposite were determined from elemental mapping analysis and the results are depicted in Fig. 3c–h. Therefore, these results have undoubtedly confirmed the homogeneous and effective formation of SRN nanocomposite, which may enhance the photocatalytic performance.

**Figure 4:**
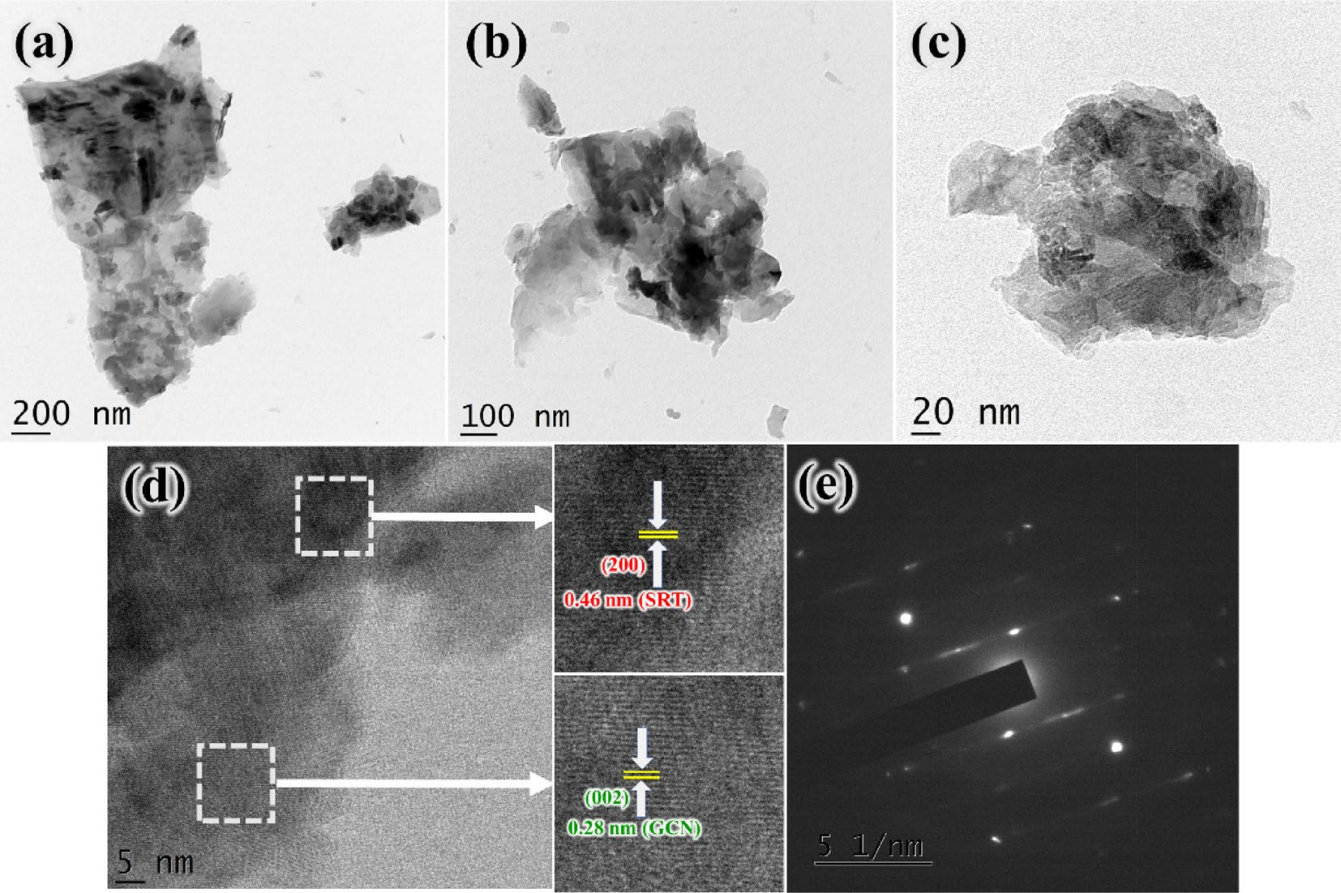
(**a**)–(**d**) HRTEM images and (**e**) SAED pattern of SRN nanocomposite.

The detailed internal nanostructures and morphology of the SRN nanocomposite were analysed by HRTEM, and the corresponding outcomes are displayed in Fig. 4a–e. As shown in Fig. 4a–c, the agglomerated SRT nanoparticles are dispersed and distributed throughout the surface of the rGO sheets and GCN nanostructures. In addition, it could be seen that the construction of this heterostructure exhibits a sandwich like formation that could enable a broad absorption spectrum with the help of rGO and GCN interlayers. Therefore, the direct contact between these ternary systems can effectively reduce the possibility of photogenerated charge carriers’ recombination and serves as a pathway for electrons to rapidly move across the heterostructure, thus leading to enhancement of photocatalytic activity ^64^. Figure 4e and its enlarged images, shows the interplanar lattice fringes to be 0.46 nm and 0.28 nm, which corresponds to (200), (002) crystalline planes of SRT and GCN samples, respectively. Besides, the bright spot ring patterns visualized from selected area electron diffraction (SAED) in Fig. 4e represents the crystalline nature. The results are well in accordance with the XRD and FTIR results.

### XPS analysis

**Figure 5:**
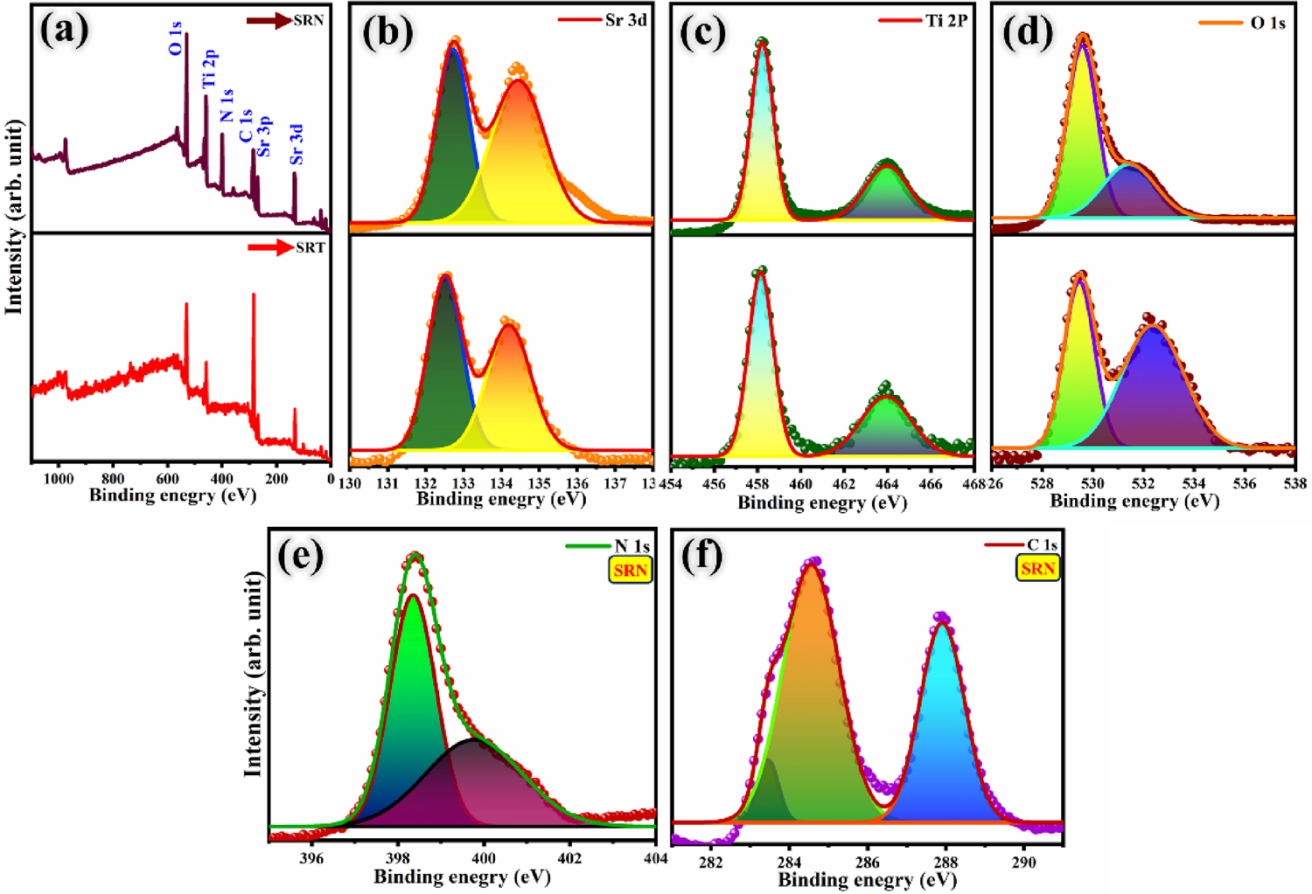
XPS spectra (**a**) survey spectra, (**b**) Sr 3d, (**c**) Ti 2p, (**d**) O 1s for SRT and SRN samples, along with (**e**) N 1s and (**f**) C 1s for SRN nanocomposite.

**Figure 6:**
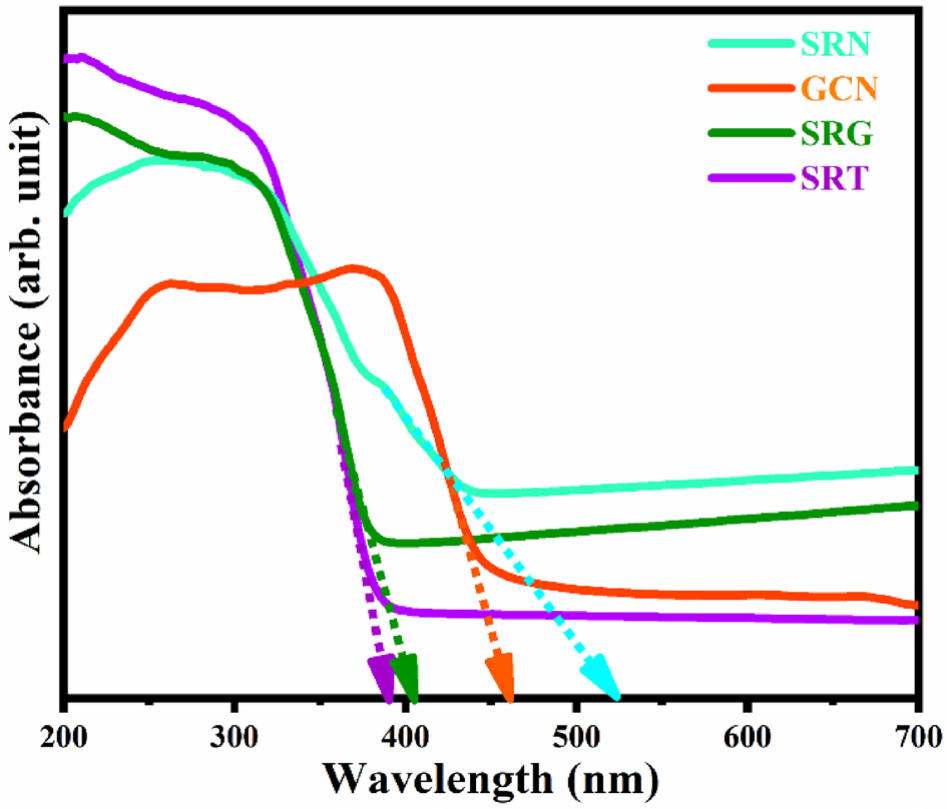
UV–Vis absorption spectra for the prepared samples.

The chemical valence state and elemental composition of SRT and SRN samples were analysed by XPS measurements. Elements like Sr, Ti, N, O and C in the pure and ternary specimens were identified and visualized in the full range of the surveyed spectrum in Fig. 5a. The high resolution XPS spectra of individual elements occurring in the specimens are clearly displayed in Fig. 6b–f. The Sr 3d peaks in Fig. 5b with 132.5 eV and 134.3 eV for SRT and peaks at 132.7 eV and 134.5 eV for SRN nanocomposite showing its binding energy positions correspond to the Sr 3d_5/2_ and Sr 3d_3/2_ states, respectively^65^. The XPS Sr 3d spectra evidenced by the dominant existence of Sr^2+^ in the synthesized samples ^66^. In the case of Ti 2p the binding energy positions indexed at 458.1 eV and 463.9 eV correspond to Ti 2p_3/2_ and Ti 2p_1/2_, respectively, for TiO_2_ with a Ti^4+^ oxidation state^67^, as shown in Fig. 5c. From Fig. 5d, the two peaks for O 1s spectra appeared in the range of 529.7 eV and 531.2 eV binding energy values refer to the presence of lattice oxygen (O_L_), oxygen vacancies, adsorbed oxygen (O_H_), and hydroxyl species on the surface^68^. The XPS results of both SRT and SRN on specific elements were similar to its deconvoluted spectra with slight changes in binding energy positions. It is obvious that the differences are ascribed due to the strong interactions of rGO and GCN on SRT sample^69^. The splitted two characteristic peaks of N 1s located at 398.6 eV and 399.6 eV, in Fig. 5e, are attributed to the occurrence of nitrogen groups in the triazine rings (C=N–C) and –NH_2_ chemical states, respectively^33,70^. From Fig. 5f, the C1s XPS spectra deconvoluted into three binding energy positions located at 283.4 eV, 284.6 eV and 288.7 eV, respectively. These peaks are attributed to N–C–N coordination of carbon and nitrogen containing groups, and adsorbed carbonates on SRN surface. Furthermore, the lattice vacancies on the surface could efficiently boost the adsorption of water induced hydroxyl groups^71,72^. Therefore, the presence of graphitic carbon nitride and carbon in the samples are evidently confirmed^73^. The above-mentioned outcomes suggest that the strong interfacial chemical bonding facilitates excellent transportation of photoproduced charge carriers, which might lead to enhancement of the photocatalytic activity.

### BET analysis

The specific surface area and pore size distribution of the SRT and SRN samples were examined using N_2_ adsorption–desorption isotherm analysis. As shown in Fig. S2, the acquired both isotherms are type III, which is the identical characteristics of mesoporous structure of the material. The obtained BET multi point surface area reveals for SRT and SRN samples, have 34.817 m^2^/g and 28.206 m^2^/g, respectively. Besides, the pore size distributions obtained from BJH method is SRT (13.64 nm) and SRN (13.78 nm). The pure SRT sample shows lower surface area, after the inclusion of rGO and GCN in SRT, the SRN ternary nanocomposite possess decreased in surface area, which is attributed due to the agglomeration and higher level of carbonaceous combinations in the composite samples^74^. Additionally, the considerable change in specific surface area and pore size distribution was occurred upon formation of the nanocomposite of semiconductors with rGO and GCN^75^. Thereby, the SRT particles exhibited with the silt/sheet-like pores of GCN and rGO, these pores could serve as evidence of a strong association between rGO/GCN and SRT. Furthermore, particle size analysis (Fig. S3) reveals average particle size of 684 nm and  ~ 324 nm for SRT and SRN samples, respectively, indicating a minimal agglomeration of the metal oxide nanoparticles even after dispersing the colloidal solution for several cycles.

### UV–Vis absorption analysis

The optical properties of the prepared samples were investigated by UV–Vis absorption analysis, and the recorded absorption spectra are presented in Fig. 6. As shown in Fig. 6, for pure SRT sample the absorption edge was exhibited at  ~ 389 nm, which shows no obvious response in the visible region due to an intrinsic bandgap in the SRT sample^76,77^. The prepared samples of SRG, GCN and SRN show their absorption edge  ~ 405, 460, and 522 nm, respectively. All these absorption band edge positions are red shifted and the optical properties are altered after being composed with GCN and rGO in SRT sample. Therefore, the shifts in absorption spectra clearly suggest that the synthesised SRN nanocomposite has an enhanced visible light absorption ability compared with the other samples. Additionally, the direct bandgaps of the prepared samples were estimated by following relation^78,79^,

1 $$\left( {\upalpha {\text{h}}\upnu } \right)^{2} = {\text{k}}\left( {{\text{h}}\upnu - {\text{E}}_{{\text{g}}} } \right)$$

where α is the absorption coefficient, E_g_ is the bandgap energy, K is a proportionality constant, and hν is the photon energy. From the above relation (1), the direct bandgap (absorption edge) was calculated, and the obtained values were 3.19 eV (389 nm), 3.06 eV (405 nm), 2.70 eV (460 nm) and 2.38 eV (522 nm) for SRT, SRG, GCN and SRN, respectively. Those results indicate that the bandgap values gradually decrease towards the visible region, which is due to the influence of carbonaceous material in the SRN nanocomposite. Moreover, the synthesised ternary nanocomposite could have an advantageous capability to harvest more visible light and it might generate more energetic photoproduced charge carriers, which enhance the photocatalytic degradation efficiency.

### Photoluminescence analysis

**Figure 7:**
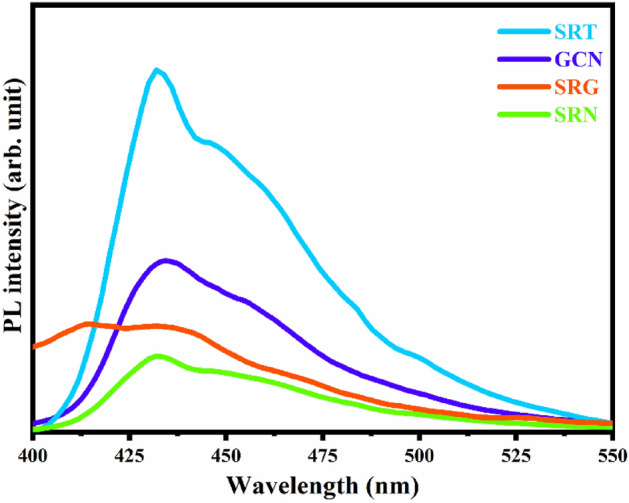
Photoluminescence spectra of SRT, SRG, GCN and SRN samples.

To understand the charge carrier recombination, migration and transfer process, the prepared samples were examined by PL spectroscopic analysis^80^. All the spectra were recorded in the range from 400 to 550 nm with an excitonic wavelength 350 nm, and their consequences are displayed in Fig. 7. The SRT sample shows its strongest emission peak at  ~ 430 nm, which is attributed to the presence of defects in the emission. The bandgap between Ti-3d conduction band and oxygen-2p valence band is  ~ 3.19 eV (Fig. 6). Furthermore, the pure GCN and SRG samples show their emission peaks with suppressed intensity compared to pure SRT, as shown in Fig. 7. All these PL spectra with strong intensity can increase the recombination rate of photoproduced e^−^/h^+^ pairs. On the other hand, weaker PL intensity facilitates the lowest recombination rate, which could enhance the photocatalytic degradation efficiency^81,82^. Figure 7 shows that upon the addition of rGO to SRT sample, the SRG’s PL spectra were quenched slightly, which could result in less light absorption and may reduce the photocatalytic performance. The addition of GCN to the SRG sample caused the PL emission peak to be ultimately quenched, which is due to strong interfacial formation and the trapping states present in the ternary nanocomposite. Thereby, the SRN photocatalyst effectively hinders the e^−^/h^+^ pair recombination rate and the charge carriers can easily migrate across the interfaces with extended lifetime. Therefore, it is evident that lower PL intensity of SRN nanocomposite, along with enhanced charge separation efficiency, would lead to achieve an enhanced photocatalytic degradation performance by improving the production of radicals in the dye eviction process^83^.

### Photocatalytic activity

**Figure 8:**
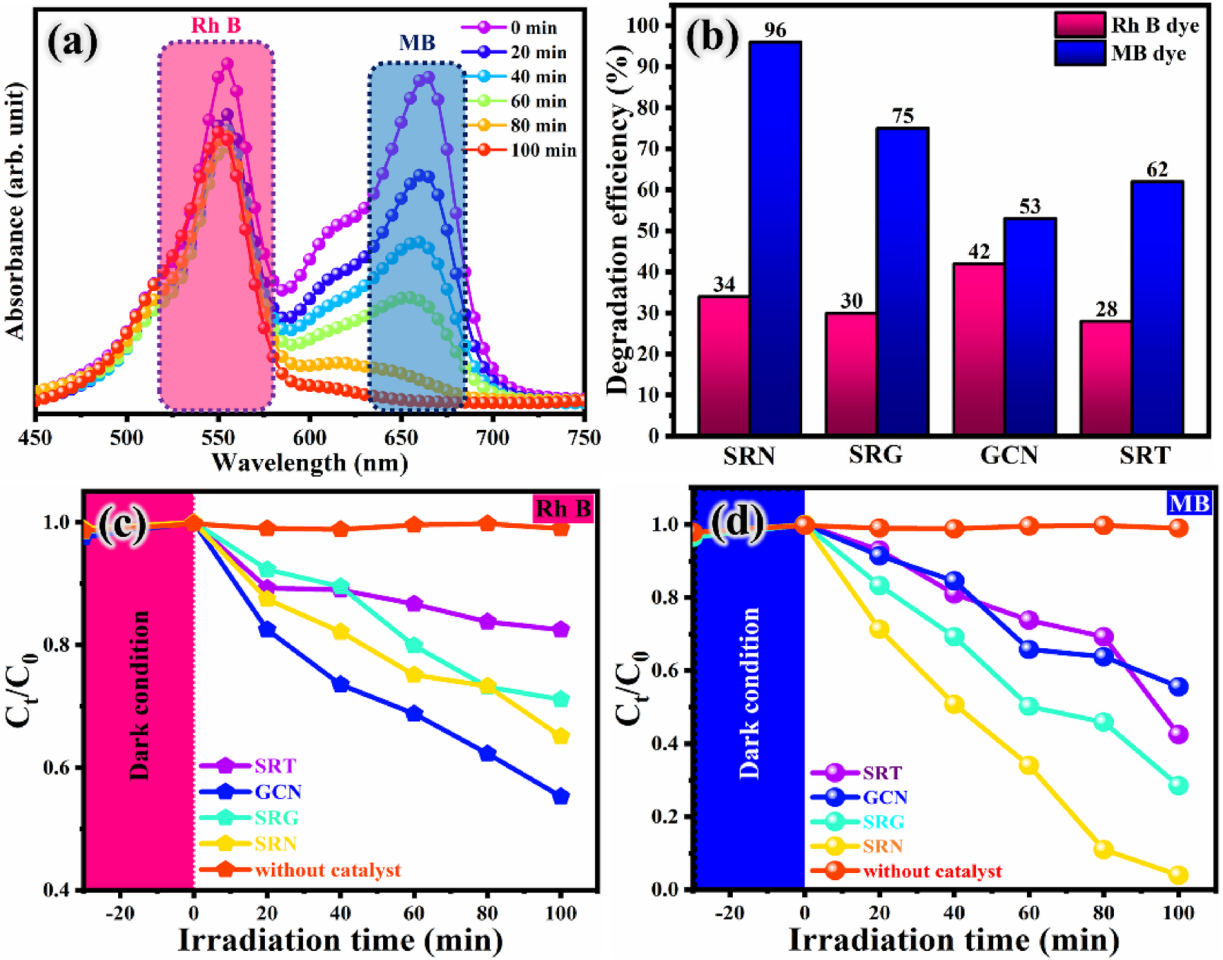
(**a**) UV–Vis absorption spectra for mixed dye degradation by SRN nanocomposite, (**b**) degradation efficiency chart and (c & d) degradation rate plot for the synthesised nanocomposites.

The photocatalytic degradation performance of the prepared specimens was investigated via representative pollutants of mixed MB and RhB dye under UV–Vis light irradiation. The concentration of dye degradation was monitored by UV–Vis spectroscopic analysis and the absorption spectra for SRN photocatalyst were plotted and presented in Fig. 8a. At first, the bare samples of SRT and GCN were tested over mixed dye and the degradation efficiency of 62%, 53% for MB, respectively and RhB degradation efficiencies were obtained as 28% for SRT and 42% for GCN sample. These outcomes showed very minimal level of dye removal concentrations. In addition, the binary combination of SRG nanocomposite exhibits 30% and 75% degradation efficiency over mixed dyes of RhB and MB, respectively. Furthermore, the enhancement in photocatalytic performance was attained by SRN nanocomposite by 96% for MB dye and 34% for RhB dye after 100 min of UV–Vis light irradiation. The calculated degradation efficiency chart is depicted in Fig. 8b. By comparison, RhB dye was not that much degraded compared to MB dye degradation performance.

The fastest degradation of MB dye by SRN nanocomposite in aqueous medium, in which the dye molecules might be easily captured with the contribution of more free charge carriers effectively promoting the electron transportation across the ternary system. Furthermore, compared to other photocatalyst the SRN nanocomposite provides ultimate degradation efficiency of MB dye, due to its higher production and ease-of-transportation of photoproduced charge carriers, as well as the maximum separation ability of photo-generated charge carriers. The highest inhibition of charge carriers effectively enhances the involvement of e^−^/h^+^ pairs in the oxidation and reduction process^84^. Notably, the photodegradation rate of the RhB dye is suppressed due to the usage of a concentrated dye in the experiment, which may cause a decrease in catalyst activity. In addition, the MB could become active with better absorption ability of the negative charges occurring on the surfaces of catalyst than RhB dye^85,86^. Moreover, the degradation processes happen by the formation of hydroxyl radicals (·OH) and super-oxide radicals (·O_2_^−^) known as reactive oxygen species (ROS). The increase in the initial concentration of the dye causes an increase in dye absorption rather than in the photocatalyst with minimum reaction of ROS, which led to a decrease in catalyst activity^87^. As shown in Fig. 8c,d, the degradation rate of the different samples was calculated, and the kinetics pertaining plots of C_t_/C_0_ versus irradiation time were plotted to evaluate the differences of photocatalytic performances.

From the photocatalytic experiment results, the linear relationship of irradiation time (t) *vs* slope of the straight-line plot (ln C_0_/C) over mixed dye degradation for different samples were calculated by ln C/C_0_ = k_app_, whereas, k—is the apparent rate constant, C_0_—concentration at 0 min, and C_t_—is the concentration at t = t.

**Figure 9:**
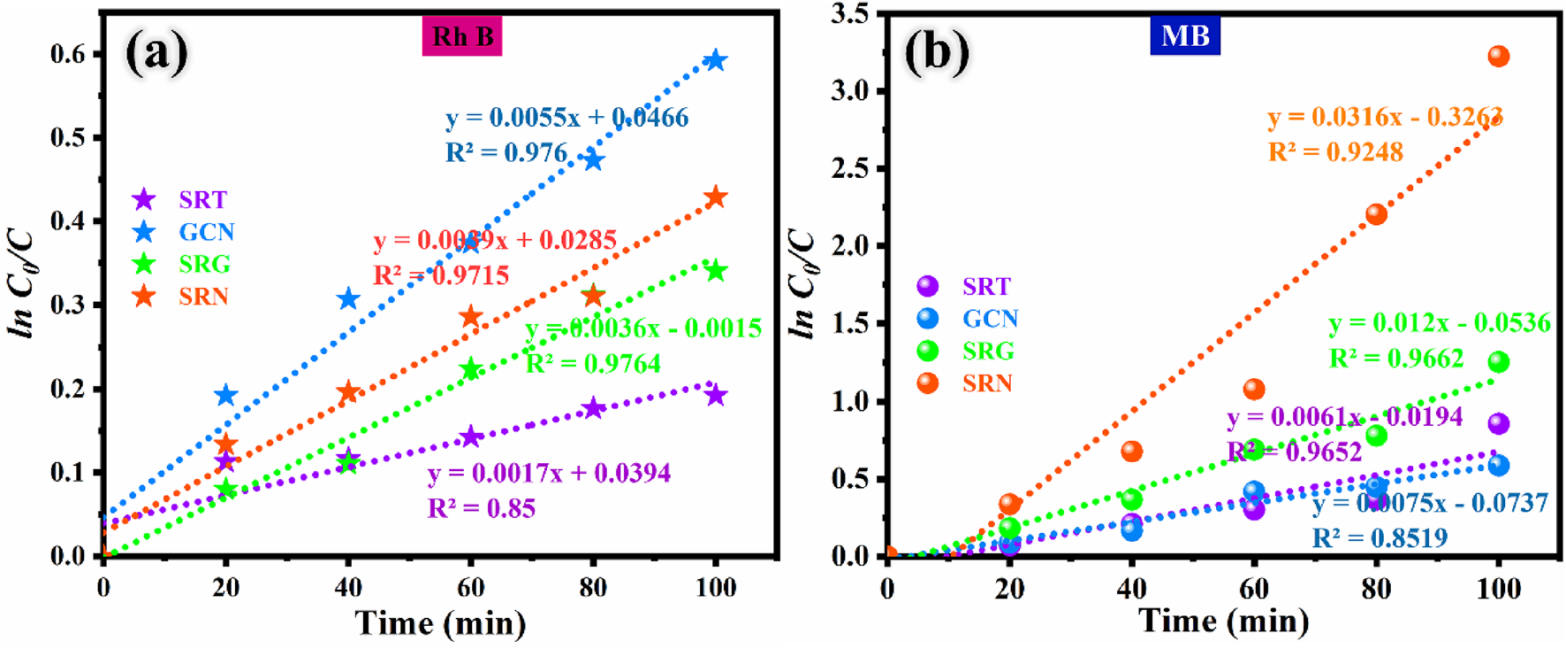
First-order kinetic plot for the degradation of (**a**) RhB dye and (**b**) MB dye over SRT, SRG, GCN and SRN photocatalysts.

The results are plotted and visualized in Fig. 9a,b. As shown in Fig. 9a,b, the derived degradation reaction kinetic processes are well obeying the pseudo first order equation function model. The photocatalytic degradation reaction curves of all the photocatalysts are well fitted with first-order (pseudo-order) kinetics. In Fig. 9a, the kinetic plot of GCN photocatalyst shows highest removal rate over RhB dye which is 3.23 folds and SRT sample over MB dye shows 2.11 times higher than other photocatalysts. As shown in Fig. 9b, the maximum dye removal rate constant was attained by SRN ternary photocatalyst over MB dye, which yields 4.21 times higher than other synthesised photocatalysts. From these results, the degradation rate of RhB dye is very low in comparison to MB dye performance of the SRN sample. The unappreciable performance of RhB dye is due to higher stability of its molecules. On the other hand, MB dye degrades easily since all the photons are absorbed, which dominates the degradation ability. The degradation rate constant and the slope of a fitting line values are obtained from Fig. 9a,b and the derived values are given in Table 1.

**Table 1:** Efficiency, K_app_, R^2^ values of the as-prepared samples.

S. no	Photocatalysts	Efficiency %		K_app_ (min^−1^)		Correlation coefficient (R^2^)	
		RhB	MB	RhB	MB	RhB	MB
1	SRT	28	62	0.0017	0.0061	0.8500	0.9652
2	GCN	42	53	0.0055	0.0075	0.9760	0.8519
3	SRG	30	75	0.0036	0.0120	0.9764	0.9662
4	SRN	34	96	0.0039	0.0316	0.9715	0.9248

### Scavenger test and effect of pH

**Figure 10:**
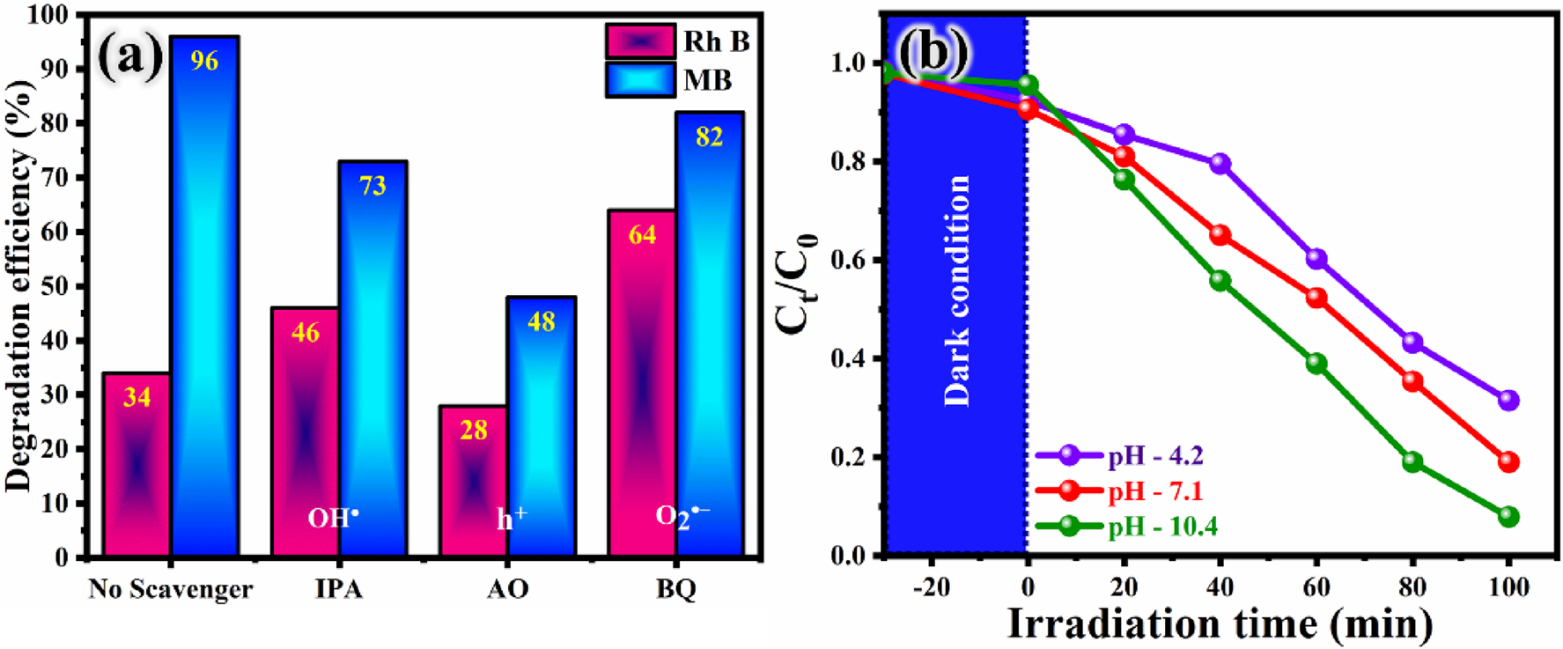
Effect of SRN photocatalyst on (**a**) different scavengers over RhB and MB dye and (**b**) different pH against MB dye degradation.

The evaluation of quenchers responsible for the photocatalytic degradation process is necessary to get insight into the degradation mechanism. The various radical trappers like benzoquinone (BQ) for O_2_^·−^, isopropyl alcohol (IPA) for OH^·^ and ammonium oxalate (AO) for h^+^ are used to identify the radicals involved in the decomposition of the dyes. The results of the radical trapping experiment in the presence of SRN photocatalyst are presented in Fig. 10a. Upon the addition of scavengers during the trapping experiment, the dye removal efficiency over the mixed dye decreases considerably, indicating that reactive species could obviously be involved in the dye decomposition reactions. In particular, the scavenger that significantly reduces the degradation efficiency percentage could play an advantageous role in the dye eviction process. As a result, when the IPA was added to the solution, the mixed dye efficiencies decreased from 96 to 73% for the MB dye, and increased by 34% to 46% for RhB, indicating that OH^·^ radicals act as an additional booster for the degradation of the RhB dye. By introducing BQ as a scavenger, the dye removal efficiency diminished from 96 to 82% for the MB dye and increased from 34 to 64% for the RhB dye, which means that the O_2_^·−^ radical also plays an advantageous role in evicting the RhB and the MB dyes. Notably, by the implementation of AO as a h^+^ quencher, the degradation efficiency percentage of both dyes are greatly suppressed from 96 to 48% for the MB dye, and from 34 to 28% for the RhB dye in the decomposition process. Among all the used scavengers, the addition of AO exhibited a predominant effect in decreasing the degradation efficiency, therefore h^+^ played an extraordinary role in the mixed dye degradation process.

Various dye industries in the society releases different pH values of waste water to the environment. pH is a crucial factor in photocatalytic degradation of various pollutants in waste water. Because it significantly affects interfacial processes, both anodic and cathodic, by altering the charge generated on the catalyst surface^88,89^. So, it is essential to examine the dye degradation performance across different pH values. To investigate the impact of pH on the degradation activity of SRN nanocomposites against MB dye, experiments were conducted at pH levels ranging from 4 to 10.5, with same procedure followed for photocatalytic experiment. The results are illustrated in Fig. 10b, providing comparative study of various strength of acidic (HCl) pH—4.2, neutral (Na_2_SO_4_) pH—7.1 and alkaline (NaOH) pH—10.4 for the experiment. The degradation efficiency was increased from 72.45% to 93.4%, by increasing the pH concentration from 4 to 10.4. The exhibited surface of the photocatalyst is negatively charged which can significantly enhance the adsorption of positively charged dye molecules, leading to higher degradation efficiency. The presence of rGO can further facilitate electron transfer, reducing recombination of electron–hole pairs and boosting overall photocatalytic activity. The surface charges SRN nanocomposite influenced at different pH levels by affecting the adsorption of dye molecules and the increasing the charge carrier separation and transfer efficiency^90^.

### Recycle and stability test

**Figure 11:**
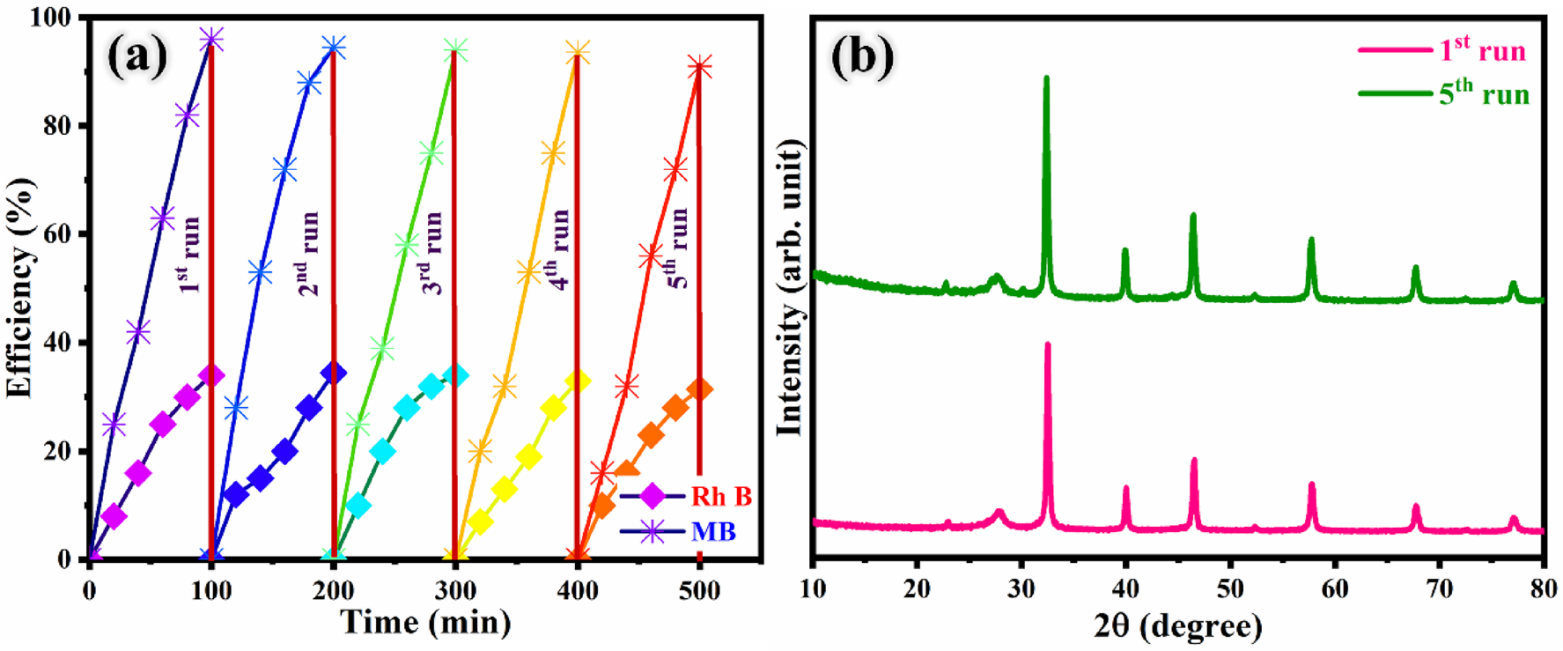
(**a**) Degradation efficiency chart for mixed dyes and (**b**) XRD pattern for 1st and 5th cycle of SRN photocatalyst.

To affirm the stability and sustainability of the photocatalyst after the photocatalytic experiment, it is essential to evaluate the stability by a recycle experiment for SRN photocatalyst. As a part of the recycle test, the SRN sample was utilized for five consecutive runs over a mixed dye degradation for 100 min under UV–Vis light irradiation, and the degradation efficiency was calculated and displayed in Fig. 11a. The highest degradation efficiency of SRN sample was slightly affected after five cycles of the recycle experiment. The change in the degradation efficiency in each cycle is attributed to catalyst loss during the recovery processes the recycle experiment. Thus, the prepared SRN photocatalyst still maintained negligible degradation efficiency, suggesting that SRN sample manifests excellent reusability. In addition, XRD analysis was performed on the photocatalyst recovered from first and fifth runs of the recycling experiment to investigate stability. The recorded patterns are shown in Fig. 11b, which clearly indicates that the structure of SRN photocatalyst was not affected and greatly sustained stability. From the above results, it is confirmed that the synthesised SRN nanocomposite is a quite stable, durable, photo corrosion resistance, and highly efficient material for real time energy and waste water treatment applications^91^. Therefore, the outcomes of SRN ternary photocatalyst exhibited outstanding performance with enhanced UV–Vis capability and improved charge separation/transfer efficiency. A comparative study of dye degradation performance with that in previously published articles is given in Table 2.

**Table 2:** Comparison study of dye degradation efficiencies by previously reported photocatalysts.

S. no	Photocatalyst	Degradation efficiency (%)	Reaction time (min)	Pollutant	References
1	ZnO/g-C_3_N_4_	~ 51	180	RhB	^92^
2	SrTiO_3_/rGO	~ 91	150	MB	^43^
3	Graphdiyne − ZnO Nanohybrids	~ 68 and 55	120	MB & RhB	^93^
4	CuO-nanozeolite X	~ 20 and 68	180	RhB + MB (Mixed dye)	^94^
5	Ag/BiVO_4_	~ 81 and 91	180	Mixed dye	^95^
6	gC_3_N_4_:rGO:ZnO-Ag	~ 83 and 90	100	Mixed dye	^61^
7	SrTiO_3_/rGO/g-C_3_N_4_ (SRN)	~ 36 and 96	100	Mixed dye	This work

### Band position and mechanism

**Figure 12:**
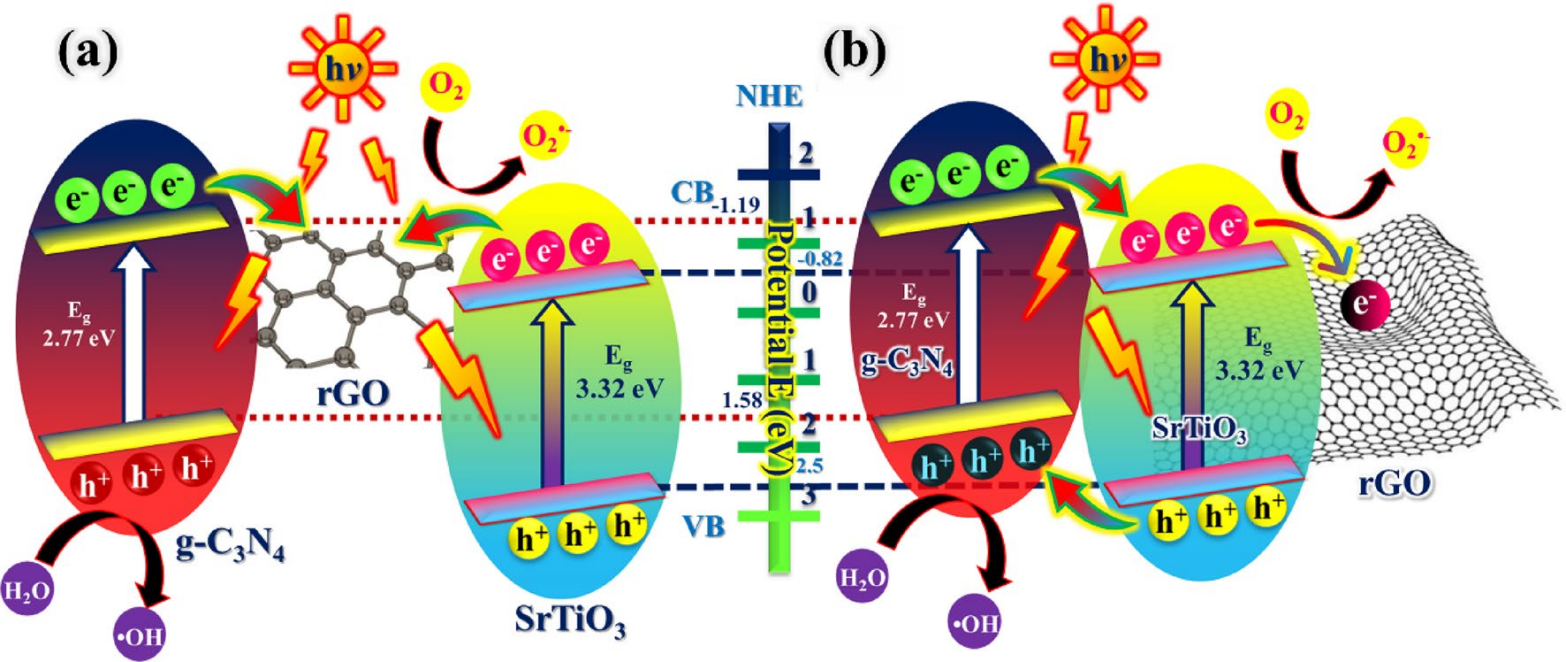
Schematic illustration of plausible degradation mechanism (**a**) rGO as intermediator and (**b**) rGO as direct contact with photocatalysts.

From the above experimental results, a plausible photocatalytic mechanism for the SRN photocatalyst and the band positions of bare samples are proposed and depicted in Fig. 12a,b. The band edge positions are estimated by Mullikan’s electronegativity theory^96^, and the absolute electronegativity values for GCN is 4.72 eV and for SRT is 5.34 eV^97,98^. Based on a similar formula reported in our previous work, the valence band (VB) and conduction band (CB) edge potentials are calculated using energy band gaps of the prepared photocatalysts^99^. All the obtained values of VB and CB positions of SRT and GCN from the calculations are elucidated in Table 2. The CB and VB positions of GCN lies at − 1.19 eV and 1.58 respectively. For the SRT photocatalyst the CB potential position is − 0.82 eV and for VB is 2.5 eV.

During photocatalytic experiments, when the UV–Vis light illuminated on the photocatalysts the photoproduced e^−^ from VB positions are excited from CB positions of both GCN and SRT samples. Hence, the holes are left behind at VB positions to form a e^−^/h^+^ pairs for in a possible degradation process. In this work, the synthesized ternary nanocomposite exhibits two possible photocatalytic charge transfer processes, as presented in Fig. 12a,b. Figure 12a shows that when rGO acts as an interlayer/junction between GCN sheets and SRT nanoparticles, the photogenic charge carrier of e^−^ at CB positions of both photocatalysts might be captured by rGO nanosheets. Thereby, the quick electron transportation and capturing by rGO network could effectively hinder the e^−^/h^+^ pair recombination and ultimately enhances the charge separation process. From Fig. 12b, if the GCN sheets directly forms a heterojunction with SRT nanoparticles in the absence of rGO as intermediator, the photogenerated e^−^ at CB of GCN can be directly transferred to the CB of SRT due to potential differences between GCN and SRT as summarized in Table 3. Therefore, the e^−^ positioned at CB effortlessly transport across the ternary system due to the formation of p–n junction between GCN and SRT. Then the e^−^ are quickly transported to catalysts surface via the conductive rGO transporting medium. Hence, due to the unique properties such as high electron mobility, and improved electrical and thermal conductivity of rGO, it can act as quick e^−^ transporter and acceptor across the medium of photocatalyst surfaces, thus extending the lifetime of charge carriers, and effectively enhancing the photocatalytic activity. Moreover, this process can lead to an efficient charge separation and inhibiting the e^−^/h^+^ pair recombination process^100^.

**Table 3:** Energy bandgap, electronegativity, VB and CB positions of the as-prepared samples.

S. no	Photocatalysts	Band gap (eV)	Electronegativity (eV)	VB (eV)	CB (eV)
1	SRT	3.32	5.34	2.5	− 0.82
2	GCN	2.77	4.72	1.58	− 1.19

During the photocatalytic degradation process, the transported photoproduced e^−^ at surface of rGO can be absorbed by O_2_ molecules to create O_2_^·−^ radicals by the photo reduction process with the h^+^ left behind at the VB position of GCN. Many abundant OH groups can directly interact with water molecules to form OH^·^ radicals by photo-oxidation process. Eventually, both the converted superoxide and hydroxyl radicals by enhanced redox reactions have durable oxidation capability to decompose the mixed dyes, which get converted into CO_2_, H_2_O minerals acids, etc. Therefore, the above possible degradation mechanism reveals a Z-scheme charge process with enhanced photocatalytic performance. Based on the outcomes of the possible degradation processes shown in Fig. 12a,b, the synthesized ternary photocatalyst possess dual charge transfer mechanism and therefore the possible reaction process is as follows:

2 $${\text{g - C}}_{{3}} {\text{N}}_{{4}} + {\text{SrTiO}}_{{3}} + {\text{hv}} \to {\text{g - C}}_{{3}} {\text{N}}_{{4}} + {\text{SrTiO}}_{{3}} \left( {{\text{e}}^{ - } + {\text{ h}}^{ + } } \right)$$

3 $${\text{g - C}}_{{3}} {\text{N}}_{{4}} + {\text{SrTiO}}_{{3}} \left( {{\text{e}}^{ - } } \right) + {\text{rGO}} \to {\text{g - C}}_{{3}} {\text{N}}_{{4}} + {\text{SrTiO}}_{{3}} + {\text{rGO}}\left( {{\text{e}}^{ - } } \right)$$

4 $${\text{rGO}}\left( {{\text{e}}^{ - } } \right) + {\text{O}}_{{2}} \to {\text{rGO }} + {\text{ O}}_{{2}}^{ \cdot - }$$

5 $${\text{g - C}}_{{3}} {\text{N}}_{{4}} + {\text{SrTiO}}_{{3}} \left( {{\text{h}}^{ + } } \right) + {\text{OH}} \to {\text{OH}}^{ \cdot } + {\text{g - C}}_{{3}} {\text{N}}_{{4}} + {\text{SrTiO}}_{{3}}$$

6 $${\text{Mixed}}\,{\text{dye}}\, + {\text{OH}}^{ \cdot } \to {\text{CO}}_{{2}} + {\text{H}}_{{2}} {\text{O}}\,\left( {{\text{decomposed}}\,{\text{other}}\,{\text{products}}} \right)$$

7 $${\text{Mixed}}\,{\text{dye}} + {\text{O}}_{{2}}^{ \cdot - } \to {\text{CO}}_{{2}} + {\text{H}}_{{2}} {\text{O}}\left( {{\text{decomposed}}\,{\text{other}}\,{\text{products}}} \right)$$

## Conclusion

A simple wet impregnation method was used to fabricate the SRN nanocomposite with a dual photocatalytic mechanism. The prepared SRN catalyst showed enhanced degradation activity against mixed dye by isolated photoproduced e^−^ and h^+^. The implementation of rGO facilitates a quicker charge transportation between SRT and GCN interfaces, so that the abundant active radical groups and effective conduction medium provides an enhanced photocatalytic activity under UV–Vis light illumination. The degradation efficiency rate of RhB and MB dyes was achieved as 0.0039 min^−1^ and 0.0316 min^−1^ of k factor value, respectively which is 4.21 (MB) and 3.26 (RhB) folds higher than other synthesized photocatalyst. The acquired SRN nanocomposite exhibited with improved photocatalytic behavior like notable photocatalytic degradation performance, excellent reusability, and stability. Moreover, the reactive OH^·^ and h^+^ radicals were dominating active species in the degradation reaction process identified from radical trapping experiment. From various analyses, the various radicals responsible for degradation process, the radical contribution for the appropriate charge dynamics, and sustainable supportive charge potentials are evaluated. Thereby, it is evident that the SRN photocatalyst, with efficient UV–Vis light active material, will be a great prospect in real-time practical applications to prevent the environmental pollution of organic dye contaminant.

### Supplementary Information

Supplementary Information.

## Data Availability

Data will be made available upon request through Dr. Venkatesh Gopal (venkeyphy@gmail.com).
