# Supplementary material for: Fabrication of SrTiO3 anchored rGO/g-C3N4 photocatalyst for the removal of mixed dye from wastewater: dual photocatalytic mechanism
Source: Sci Rep. 2024 Jul 15;14:16259. doi: 10.1038/s41598-024-66844-x (PMC11251063; doi:10.1038/s41598-024-66844-x)
Supplement: Supplementary file 1 — Supplementary Information. [file 41598_2024_66844_MOESM1_ESM.docx]

**Supporting information**

**Fabrication of SrTiO_3_ anchored rGO/g-C­_3_N_4_ photocatalyst for the removal of mixed dye from wastewater: Dual photocatalytic mechanism**

Venkatesh Gopal^a^*, Govindasamy Palanisamy ^b^*, , Jintae Lee^b^, Imad A. Abu-Yousef^a^, Amin F. Majdalawieh^a^, Amjad Mahasneh^a^, Kattupatti M. Prabu^c^, Sofian Kanan^a*^

*^a^ Department of Biology, Chemistry & Environmental Sciences, College of Arts and Sciences, American University of Sharjah, Sharjah P.O. Box 26666, United Arab Emirates.*

*^b^* *School of Chemical Engineering, Yeungnam University, 280 Daehak-Ro, Gyeongsan, 38541, Republic of Korea.*

*^c^ PG & Research Department of Physics, Sri Vidya Mandir Arts & Science College, Katteri, Uthangarai – 636 902, Tamilnadu, India*

***^*^Corresponding authors:*** *Venkatesh Gopal (*[*venkeyphy@gmail.com*](mailto:venkeyphy@gmail.com)*), Govindasamy Palanisamy (*[*palanisamyg@yu.ac.kr*](mailto:palanisamyg@yu.ac.kr)*), Sofian Kanan (*[*skanan@aus.edu*](mailto:skanan@aus.edu)*)*


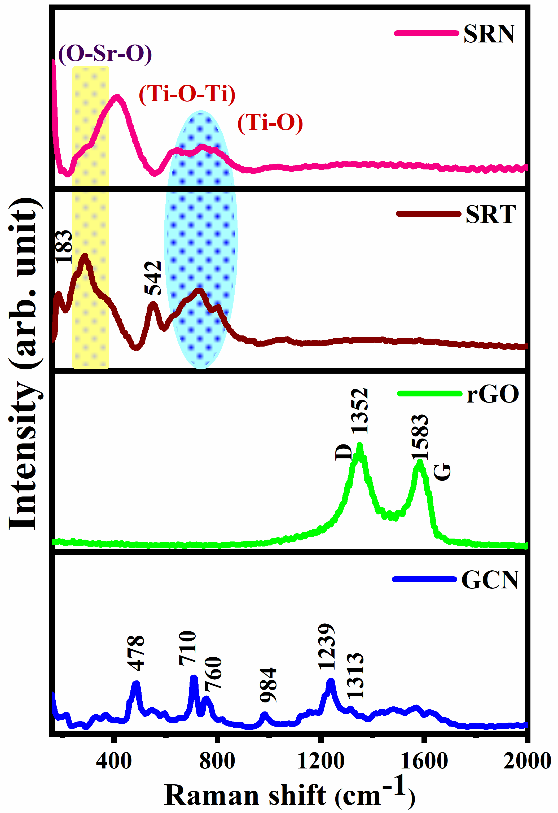


***Fig. S1. Raman spectra for GCN, rGO, SRT and SRN nanocomposite.***


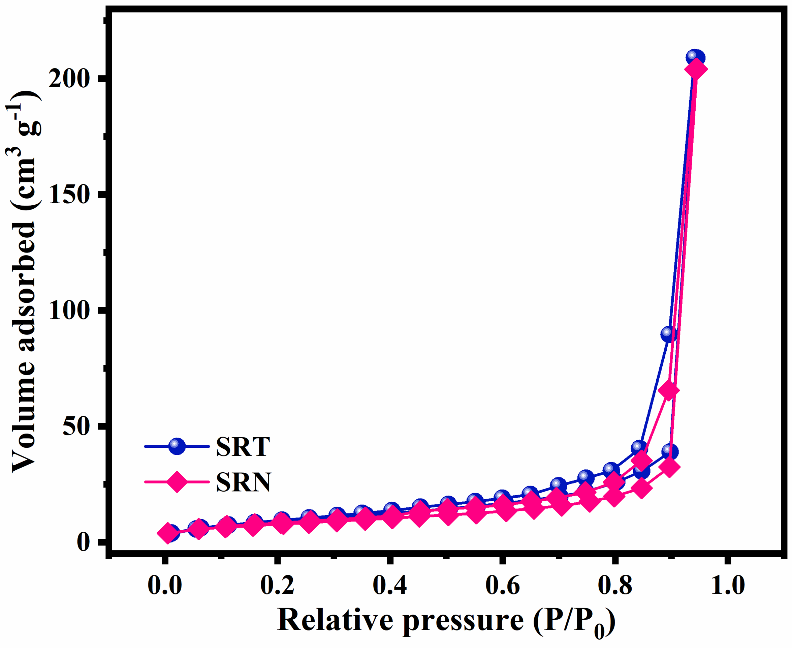


***Fig. S2. Nitrogen adsorption-desorption BET isotherms of as prepared SRT and SRN nanocomposite.***

**Particle size measurement**

The hydrodynamic size of SRT and SRN photocatalysts were examined using dynamic light scattering (DLS) particle size analyzer instrument. The hydrodynamic diameter of SrTiO_3_ inorganic core consists of metal, as well as the functional groups of integrated carbonaceous samples rGO and g-C_3_N_4_ were adsorbed onto the surface of the SRN nanocomposite. From, the Fig. S3(a & b), provides particle distribution of SRT and SRN nanocomposite, respectively, As from Fig. S3(a & b), the resulting average particle size of SRT and SRN samples are found to be ~684 nm (SRT) and ~324 nm (SRN). From the results, it can be concluded that there is minimal agglomeration of the metal oxide nanoparticles even after dispersing the colloidal solution for several periods, thereby its evidenced that the synthesized SRN nanocomposite possess exceptional stability.


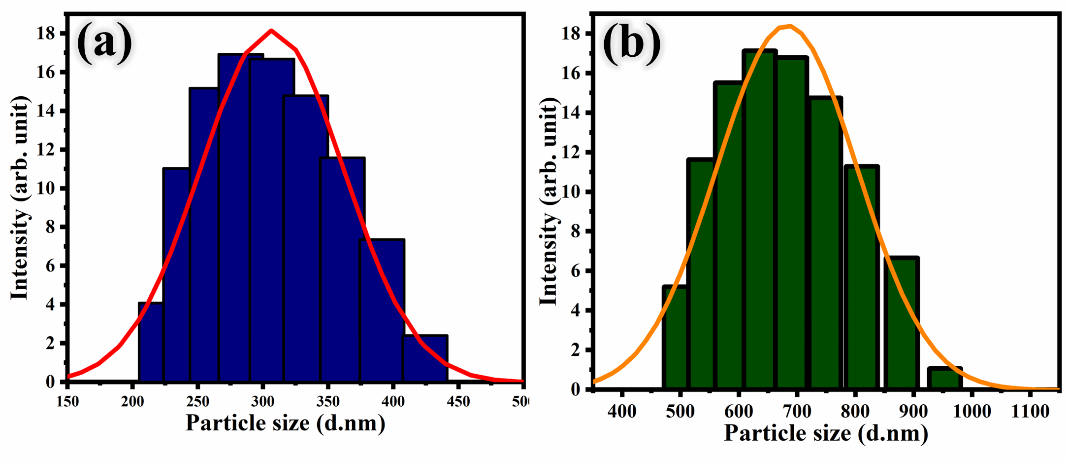


***Fig. S3. Dynamic light scattering (DLS) analysis for particle size and distribution (a) SRT and (b) SRN nanocomposite.***
